# Disrupted parahippocampal and midbrain function underlie slower verbal learning in adolescent-onset regular cannabis use

**DOI:** 10.1007/s00213-019-05407-9

**Published:** 2019-12-09

**Authors:** Grace Blest-Hopley, Aisling O’Neill, Robin Wilson, Vincent Giampietro, Sagnik Bhattacharyya

**Affiliations:** 1 Department of Psychosis Studies, Institute of Psychiatry, Psychology & Neuroscience, King’s College London, De Crespigny Park, London, SE5 8AF UK; 2 Department of Neuroimaging, Institute of Psychiatry, Psychology & Neuroscience, King’s College London, London, UK; 3 South London and Maudsley NHS Foundation Trust, Denmark Hill, Camberwell, London, UK

**Keywords:** Verbal learning, Memory, Cannabis use, fMRI, Midbrain, Parahippocampal gyrus, BOLD

## Abstract

**Rationale:**

Prolonged use of cannabis, the most widely used illicit drug worldwide, has been consistently associated with impairment in memory and verbal learning. Although the neurophysiological underpinnings of these impairments have been investigated previously using functional magnetic resonance imaging (fMRI), while performing memory tasks, the results of these studies have been inconsistent and no clear picture has emerged yet. Furthermore, no previous studies have investigated trial-by-trial learning.

**Objectives:**

We aimed to investigate the neural underpinnings of impaired verbal learning in cannabis users as estimated over repeated learning trials.

**Methods:**

We studied 21 adolescent-onset regular cannabis users and 21 non-users using fMRI performed at least 12 h after last cannabis use, while they performed a paired associate verbal learning task that allowed us to examine trial-by-trial learning. Brain activation during repeated verbal encoding and recall conditions of the task was indexed using the blood oxygen level-dependent haemodynamic response fMRI signal.

**Results:**

There was a significant improvement in recall score over repeated trials indicating learning occurring across the two groups of participants. However, learning was significantly slower in cannabis users compared to non-users (*p* = 0.032, partial eta-squared = 0.108). While learning verbal stimuli over repeated encoding blocks, non-users displayed progressive increase in recruitment of the midbrain, parahippocampal gyrus and thalamus (*p* = 0.00939, partial eta-squared = 0.180). In contrast, cannabis users displayed a greater but disrupted activation pattern in these regions, which showed a stronger correlation with new word-pairs learnt over the same blocks in cannabis users than in non-users.

**Conclusions:**

These results suggest that disrupted medial temporal and midbrain function underlie slower learning in adolescent-onset cannabis users.

**Electronic supplementary material:**

The online version of this article (10.1007/s00213-019-05407-9) contains supplementary material, which is available to authorized users.

## Introduction

The long-term use of cannabis has long been associated with deficits in cognition (Grant et al. 2003; Meier et al. 2012; Schreiner and Dunn 2012) including learning and memory function (Meier et al. 2018; Schoeler and Bhattacharyya 2013; Solowij and Battisti 2008), particularly verbal learning (Schoeler et al. 2016a). Consistent with this, acute experimental studies have demonstrated that a single dose of delta-9-tetrahydrocannabinol (THC), the main psychoactive ingredient of cannabis, or THC-rich cannabis extract, can impair memory (Curran et al. 2002; D'Souza et al. 2004) in cannabis users (CU) and alter the memory related engagement of medial temporal and prefrontal regions (Bhattacharyya et al. 2012; Bhattacharyya et al. 2009; Bossong et al. 2012) that are key to memory processing (Buckner et al. 1999; Zeineh et al. 2003). Although studies in regular CU have fairly consistently shown impairment in memory performance (Grant et al. 2003; Schreiner and Dunn 2012), evidence regarding the neurobiological underpinnings of impairments in memory following long-term cannabis use has been much less consistent (Batalla et al. 2013; Martin-Santos et al. 2010).

Previous studies have investigated brain functional activation differences between long-term CU and non-users (NU) during the performance of a range of memory tasks using functional magnetic resonance imaging (fMRI) (Carey et al. 2015; Jager et al. 2010; Jager et al. 2006; Jager et al. 2007; Nestor et al. 2008; Schweinsburg et al. 2011). Five of those studies employed associative memory tasks (Carey et al. 2015; Jager et al. 2010; Jager et al. 2007; Nestor et al. 2008; Schweinsburg et al. 2011), four used spatial memory tasks (Kanayama et al. 2004; Schweinsburg et al. 2008b; Sneider et al. 2013; Tervo-Clemmens et al. 2018) and two used working memory tasks (Jager et al. 2010; Jager et al. 2006). Performance across the memory tasks was either similar between groups (Jager et al. 2010; Jager et al. 2006; Jager et al. 2007; Kanayama et al. 2004; Schweinsburg et al. 2008b; Schweinsburg et al. 2011), or showed modest differences (Carey et al. 2015; Nestor et al. 2008; Sneider et al. 2013), with CU showing worse performance*.* No consistent pattern of functional alterations has emerged across these studies which examined different functional domains of memory using different cognitive tasks, with some showing opposite patterns of mediotemporal (Carey et al. 2015; Nestor et al. 2008; Sneider et al. 2013) and prefrontal activation (Jager et al. 2010; Jager et al. 2007; Kanayama et al. 2004; Schweinsburg et al. 2008b; Sneider et al. 2013). Of the 3 studies investigating associative learning in adults (Carey et al. 2015; Jager et al. 2007; Nestor et al. 2008), Carey et al. investigated activation during the learning condition of a location-number association task that correlated with corrected or repeated errors during a subsequent recall condition, rather than learning or encoding of new information per se (Carey et al. 2015). Using a functionally defined region of interest analysis (ROI) approach that identified the ROI regions from thresholded error-related activations in all subjects, they found decreased anterior cingulate and hippocampal activation in CU compared to NU. The other two studies employed a similar analysis approach in addition to also using anatomically defined ROIs to investigate brain activation while learning associations across all learning trials (Jager et al. 2007; Nestor et al. 2008). They reported significantly lower activation bilaterally in the superior frontal and in the middle frontal and superior temporal gyri on the right side (Nestor et al. 2008) and right dorsolateral prefrontal cortex and parahippocampal/ fusiform gyrus bilaterally (Jager et al. 2007), suggesting lower dorsolateral prefrontal activation in CU across these studies. Analyses using anatomically defined ROIs (hippocampal/parahippocampal regions) from these studies however revealed opposite patterns of right parahippocampal activation during the learning condition (Jager et al. 2007; Nestor et al. 2008), with Nestor and colleagues reporting greater parahippocampal activation in CU compared to NU and Jager et al. reporting an opposite effect. In their analysis of functionally defined ROIs, Jager et al. also found anterior cingulate hypoactivation during recall in CU compared NU, whereas Nestor et al. found no group difference in recall-related activation (Jager et al. 2007; Nestor et al. 2008). As neither study reported significant group differences in task performance, and both investigated comparable CU and NU groups in terms of other substance use and employed comparable learning tasks, these opposite patterns of parahippocampal activity may be attributable to the longer abstinence requirement in the Jager et al. study (at least 7 days) confirmed by urine toxicology compared to the study by Nestor and colleagues, where participants reported use upto 3 h before scanning and tested positive for THC on urine screening. Neither of the two studies investigating associative learning in adolescent CU (Jager et al. 2010; Schweinsburg et al. 2011) reported any significant difference associated with cannabis use, using either functional or anatomical ROIs, though they examined CU after a longer period of abstinence than the adult studies, suggesting that both differences in participant age and duration of abstinence may underlie lack of a consistent pattern of results, as also evident from of pooled analyses of previous neuroimaging data (Blest-Hopley et al. 2018a, b).

Both experimental administration of THC (D’Souza et al. 2004) and regular use of cannabis (Nestor et al. 2008; Solowij et al. 2002) have been shown to be associated with impaired learning over repeated trials. Previous studies also suggest that cannabis use is associated with a considerably worse effect on cognition if initiated during adolescence (Schweinsburg et al. 2008a), when the brain may be particularly sensitive to the detrimental effects of cannabinoids (Quinn et al. 2008; Schneider and Koch 2003). While previous studies reviewed above have employed paired associate learning tasks to investigate the neural correlates underlying impaired memory of associations in cannabis users, all of them examined the neural correlates of learning during the encoding blocks independent of repetition. Such an analysis approach does not allow an investigation of trial-by-trial encoding and updating of contextual information, which clearly occurs over repeated trials as evident from the progressive improvement in performance both in cannabis users and non-users (Nestor et al. 2008). However, none of the studies to date have specifically compared the learning curves and associated pattern of functional brain activation using a trial-by-trial analysis approach, to investigate the whether the gradient of learning and its neural correlates differ in cannabis users compared to non-users. Therefore, in the present study, we aimed to complement current understanding by investigating the neurophysiological correlates of impaired verbal learning in the context of adolescent-onset regular and current heavy adult cannabis use by examining the change in brain activation over repeated learning trials. As the medial temporal cortex, particularly the parahippocampal gyrus is involved in the processing of contextual and relational information (Davachi 2006; Ranganath et al. 2004); that results in subsequent successful recall, we expected that healthy non-users will show an incremental pattern of learning-related engagement of the medial temporal cortex over successive encoding trials in parallel with progressive improvement in subsequent recall score. In contrast, we predicted that cannabis users will have a slower learning trajectory and disruption in the normal pattern of learning-related incremental engagement of the medial temporal cortex. The psychotropic effects of inhaled THC, the main psychoactive cannabinoid in recreationally used cannabis, typically do not last longer than a few hours (~ 3 h) (Grotenhermen 2003). We were specifically interested in investigating the residual effects of cannabis on learning-related brain function that persist beyond the immediate acute intoxication period, rather than the long-term effects that persist even after a sustained period of abstinence.

## Methods and materials

### Participants

Twenty-two current CU (13 males, 9 females; age 24.95 ± 3.56), who had started using cannabis regularly before the age of 18, and 21 sex (12 males, 9 females) and age (–24.24 ± 4.11 years) matched NU were recruited using local and targeted online advertising. Inclusion criteria required them to have been consuming cannabis on at least four or more days per week, for the 2 years prior to taking part in the study. They should also have been regular users before the age of 18, defined as using more than twice a month and at least 10 times in their lifetime (Sznitman et al. 2015). NU control group inclusion required lifetime use of cannabis of less than 10 times before taking part in the study (Sznitman et al. 2015). Common exclusion criteria for both groups included history of a neurological disorder, diagnosis of a mental illness, being a recipient of psychiatric services, family history of psychosis in a first-degree relative and educational attainment suggestive of an IQ of less than 70, or any contradiction to MRI safety. Absence of mental illness was confirmed based on self-report. All participants in the study underwent a screening interview wherein they were asked about personal or family history of suffering from or receiving help/treatment for mental disorders. Participants were also asked whether they were on any medication. Only those who did not have a personal history (except for cannabis use disorder in the CU group) or family history of mental disorder and were not on any treatments for mental disorder, based on self-report were included into the study. We did not carry out formal IQ testing, but collected information on educational attainment and number of years in full-time education as a proxy of IQ, which has been found to correlate with educational attainment (Batty et al. 2007; Colom et al. 2002). All participants recruited to the study had qualified up to GCSE level (a secondary school leaving examination in the UK taken usually at 16 years of age or equivalent level of education), with the majority also having qualified up to A-levels (a qualification obtained following a further 2 years of full-time education and used often as a criterion for university entry) or above. All participants underwent a urine drugs screening (amphetamine, cocaine, opiates, THC, phencyclidine, benzodiazepines, barbiturates, methadone, propoxyphene) on the day of MRI scanning. NU were required to have a negative result for all substances; CU were required to have a positive result for THC, and a negative urine test result for all other drugs. Participants were asked to refrain from using cannabis or alcohol on the day of scanning, from caffeine intake for 4 h, and from tobacco use for 2 h before the scan. None of the CU reported smoking cannabis on the day of the scan, meaning all CU would have had at least 12 h of abstinence from cannabis by the time of scanning. While determining the number of subjects to recruit, we were guided by sample sizes of comparable previous studies carried out in adults, which generally examined around 14–20 participants per group (Carey et al. 2015; Jager et al. 2007; Nestor et al. 2008). We have also reported post-hoc achieved power, which we estimated (G-POWER (Faul et al. 2007)) using the effect-size estimate from the parahippocampal/midbrain cluster observed to be differentially engaged in the two groups over the repeated encoding trials, the main focus of interest for the present study.

Participants completed an adapted version of the Cannabis experience questionnaire (Barkus et al. 2006) which was used to collect information on previous cannabis and other drug exposure.

Ethical approval for the study was obtained from the King’s College London Research ethics committee (PNM RESC HR-15/16–2416). All participants provided written informed consent and were financially compensated for their time and expenses.

### Verbal paired associative learning task

Participants completed a verbal paired associate (VPA) learning task while inside the MRI scanner, as previously employed to investigate the acute effects of THC in healthy volunteers and described in detail here (Bhattacharyya et al. 2009). The task comprised encoding and recall conditions, which were compared against a baseline condition. Stimuli were presented in blocks, following a visual prompt giving instruction for the condition, as used in a previous study (Bhattacharyya et al. 2009). For the encoding condition, the visual prompt was ‘Do these words go well together’ and participants were asked to say ‘yes’ or ‘no’ after being presented pairs of related and unrelated words printed on blue rectangular boxes. For the recall condition, the visual prompt was ‘Which word was associated with this word?’ and participants were presented with one of the words (cue) from the pairs that they had been shown during the preceding encoding condition printed similarly on blue rectangular boxes. They were asked to say the word that had been presented together with the cue word in the encoding block. Each encoding condition had eight pairs of words and the recall condition had eight cue words presented for 5 s each; each encoding and recall block was repeated four times to allow an estimation of learning over repeated trials based on subsequent recall. For the low-level baseline condition, blank boxes were shown in repeated presentation in the same configuration as in the previous four conditions. All verbal responses were recorded, and only activation from correct responses was used in the analysis. Before entering the scanner, the task was explained and participants completed a practice run, using words different from the main task.

### Image acquisition and analysis

Images were acquired on a GE SIGNA HDx 3.0T MR scanner system (GE healthcare Milwaukee, USA) at the Centre for Neuroimaging Sciences, King’s College London. T2*-weighted images were acquired axially in 39 slices (3 mm) with a 0.3-mm slice gap (matrix size 64 × 64 voxels, in-plane voxel size 3.75 × 3.75 mm). A 30-ms echo time, 90° flip angle and compressed acquisition with a 2-s repetition time and 3 s silence were also used. A high-resolution gradient echo image was acquired for co-registration and to help map the fMRI data onto standard space with 43 × 3 mm slices with a 0.3-mm slice gap (matrix size 128 × 128 voxels, in –plane voxel size 1.875 × 1.875 mm). A 30-ms echo time, 90° flip angle and repetition time of 3 s were used.

fMRI data was analysed with the XBAM_v4.1 software, a non-parametric data analysis package using previously detailed approaches (Bhattacharyya et al. 2009). The non-parametric approach minimises assumptions about the distribution of the data, employing permutation rather than normal theory based inference. This is important in fMRI analysis because the distribution of data may not necessarily follow a normal Gaussian distribution (Brammer et al. 1997; Thirion et al. 2007). By using medians rather than averages as a test statistic, XBAM is less sensitive to the effects of outlier values that may bias the distribution of the data (Hayasaka and Nichols 2003). The test statistic in this method is computed by standardizing for individual differences in residual noise before embarking on a second- level, multi-subject testing, using robust permutation-based methods, employing a mixed-effects approach to deal with the issue of non-normality. The use of a mixed-effects approach addresses the issue of inequality of individual residual variances by effectively ‘down weighting’ responses with large residual variances. The significance of the resulting reweighted responses at group level is then tested by data permutation to avoid assumptions of normality.

### Individual subject fMRI analysis

The VPA task did not start until after the first four volumes were recorded. Data for the first four (dummy) fMRI volumes recorded were not used for analysis to ensure steady state magnetisation. The fMRI data was processed to minimise motion related artefacts. Images were first realigned to correct for head motion (Bullmore et al. 1999a). Head movement correction involved the computation of a 3D volume consisting of the average intensity at each voxel over the whole experiment, which was used as a template. The 3D image volume at each time-point was then realigned to this template by computing the combination of rotations (around the *x*, *y* and *z* axes) and translations (in *x*, *y* and *z*) that maximised the correlation between the image intensities of the volume in question and the template 3D volume (rigid body registration). Following realignment, the data was then smoothed by the application of an 8.8 mm full-width-at-half-maximum Gaussian filter to average the relative intensities of neighbouring voxels and to increase the signal-to-noise ratio. Slice timing correction was applied and the residual effects of motion were regressed out from the time series (using the estimated motion parameters) before fitting a general linear model.

To model the blood oxygen level-dependent haemodynamic (BOLD) response signal, the experimental design was convolved with 2 gamma-variate functions, peaking at 4 and 8 s to allow for variability in haemodynamic delay. Then, using the constrained BOLD effects model, a best fit between the weighted sum of these convolutions and the change over time at each voxel was computed (Friman et al. 2003). This step reduced the possibility of the model-fitting procedure giving rise to mathematically plausible, but physiologically implausible results. Following the least squares fitting of this model to the data, the sum of squares (SSQ) ratio (ratio of the SSQ of deviations from the mean image intensity due to the model component over the whole-time series to the SSQ of deviations due to the residuals) was estimated for each voxel, for each block and condition. Data were permuted by the wavelet-based method described and characterized previously (Bullmore et al. 2001), which permits data-driven calculation of the null distribution of SSQ under the assumption of no experimentally-determined response. This distribution can then be used to threshold the activation maps at any desired type 1 error rate. Activated voxels were then grouped into clusters using a previously described method (Bullmore et al. 1999b), which has been shown to give excellent cluster-wise type I error control. Briefly, clusters were defined as groups of significant voxels that were spatially contiguous in three dimensions. For each randomisation (*n* = 50), the sum of voxel statistics within each cluster was computed, which were combined to form an overall distribution of cluster mass under the null hypothesis. We then calculated the number of clusters that would be expected by chance alone in the randomised data in order to assess the statistical significance at the cluster level. The cluster-level *p* value was then set at a threshold for significance that provided the expected number of false positive clusters to be less than one.

The sum of squares (SSQ) ratio maps for each individual at each separate block and for each condition obtained as above were transformed into standard stereotactic space (Talairach and Tournoux 1988) using a two-stage warping procedure (Brammer et al. 1997). As a first step, an average image intensity map for each individual at each separate block over the course of the experiment was computed (i.e. realignment target used above). We then computed the transformations required to map this image to the structural scan for each individual and then from ‘structural space’ to Talairach space. The SSQ ratio and BOLD effect-size maps were then transformed into Talairach space using these transformations. The active task conditions, encoding and recall, were modelled against a low-level baseline (hereafter called as baseline) condition. Each block was modelled separately, such that activation maps were created for each condition, at four time-points, as well as across all encoding or recall blocks independent of repetition.

### Group level analysis

Group activation maps were created for the four blocks (either Encoding minus baseline or Recall minus baseline) for each group (CU and NU) by determining the median SSQ ratio at each voxel (over all individuals) in the observed and permuted data maps. Medians were used to minimise outlier effects. The distribution of median SSQ ratios over all intracerebral voxels from the permuted data was then used to derive the null distribution of SSQ ratios. This allowed group activation maps to be thresholded at the desired voxel or cluster-level type 1 error rate. The voxel-wise statistical threshold was set at *p* = 0.05 and the cluster-wise thresholds were adjusted to ensure that the number of false positive clusters per brain would be < 1 (only regions that survived this critical statistical threshold are reported). By conducting analyses at the cluster-level, data from more than one voxel is integrated into the test statistic giving greater sensitivity and allowing for a reduction in the search volume and of the overall number of required tests for whole-brain analysis. In comparison to analysis at the voxel level, cluster-level analyses thereby help to mitigate the multiple comparisons problem.

### Brain activation across all encoding and recall blocks: group comparison between cannabis users and non-users

As our main hypotheses were related to repetition-dependent change in activation during the encoding and recall conditions of the task, we have described these analyses and results in detail (below). However, for the sake of completeness, we also analysed group differences in brain activation independent of repetition during the encoding and recall conditions and these methods and results are summarised in the Online Resource 1 & 2.

### Learning over repeated encoding and recall blocks

An analysis of variance was then carried out for each group separately, looking at progressive activation increase and decrease over the four time-points of the each of the task conditions of interest (encoding and recall). For the between group analysis, we conducted a split-plot analysis of variance consisting of non-repeated measures in the first dimension (group, 2) and repeated measures in the second dimension (time-points, 4). Whole-brain analyses of variance were carried out for encoding and recall separately. For these analyses, the voxel-level statistical threshold was set at 0.05 and the cluster-level threshold was adjusted to yield less than one false positive 3D cluster per map.

To examine whether brain activation differences observed were due to successful learning of new word-pairs, we first created for each subject a ‘new learning score’ curve, which represented the number of new word-pairs successfully encoded and recalled in each encode and recall pair of blocks. This was computed by calculating for each pair of encoding and recall blocks, the number of ‘new’ word-pairs correctly encoded and recalled successfully for the first time. Subsequently, we conducted whole-brain correlational analyses between the median SSQ ratio at each voxel in each group for each subject over the four separate encoding or recall blocks with the ‘new learning score’ curve for that subject. These analyses were conducted for encoding and recall conditions separately using the same ‘new learning score’ curve created as described above. We estimated the Pearson’s product moment correlation coefficient at each voxel, yielding one correlation coefficient (r) per intracerebral voxel. Group differences in correlation were estimated at each voxel by computing, for each group independently, the r for each subject at each block and then by subtracting the resulting two values. An appropriate null distribution was then generated by randomly permuting subjects and their ‘new learning score’ performance between the groups (without replacement), therefore scrambling any group differences. For each of the many permutations, the difference in correlation between the scrambled groups was calculated as above and the resulting values were combined over all voxels to produce a whole-brain null distribution of differences in correlation. The cluster probability under the null hypothesis was chosen to set the level of expected type I error clusters to less than 1 error cluster per whole brain.

### Analyses examining the confounding effects of other drug use

To control for the potential effects of other drugs, we also covaried for other drug use in the trial-by-trial learning group level analyses during encoding and recall. A reliable way to classify other drug use is to use information on pattern of use in the last year and last month, as this serves to better define people as current, occasional or regular users (Shiner and Newburn 1997). We conducted separate analyses covarying for the effects of cocaine, MDMA, hallucinogen and nicotine use, the drugs with the most usage history in our study population. For participants reporting cocaine, MDMA or hallucinogen use, those who reported no use of the substance in the past year were coded as 0, those who reported use of a few times in the last year as 1 and those who used once or twice a month as 2. These three categories for other drug use were employed for cocaine, MDMA or hallucinogen use, separately to examine whether exposure to these drugs at either low or high levels of use may have confounded the association between cannabis use and brain activation. Subsequently, we also completed a further analysis including cocaine, MDMA and hallucinogen use in the last year together, such that there was no drug use in the last year (0), use of any of those drugs a few times in the last year (1) and any drug use once or twice a month (2). For nicotine, participants were marked as smokers (1) or non-smokers (0) and covaried in a separate analysis. Analyses were conducted as previously described for both learning and recall condition separately for each of the four drugs as covariates and together as explained above. Analyses were thresholded with a voxel *p* < 0.05 and, as before, the cluster threshold was adjusted to yield less than one false positive 3D cluster per map.

## Results

One participant from the CU group was not included in the analyses as they did not complete the first half of the task correctly. Therefore, we could not model their brain activation over the four time-points. Demographic characteristics for the subjects used in the analysis (21 per group) are shown in Table 1. CU participants used cannabis regularly (Sznitman et al. 2015) before the age of 18; where all used at least once a week except one, who reported using several times monthly. At the time of participation in the study, CU where using cannabis on average 6.19 (SD = 1.20) days per week.

**Table 1:** Socio-demographic and recent drug use history

	Cannabis users	Non-users	Statistics
Participants (*n*)	21	21	
Males (*n*)	13	12	*t* = 0.126, *p* = 0.9
Right-handed (*n*)	18	20	Fisher’s exact = 0.606
Age (mean ± SD, years)	24.95 ± 3.56	24.24 ± 4.11	*t* = − 0.602, *p* = 0.55
Age (range)	(18–34)	(19–33)	
Years of education (mean ± SD)	15.76 ± (2.07)	16.86 ± 1.24	*t* = 2.081, *p* = 0.044
Cannabis, alcohol and nicotine use descriptive			
Age of onset (mean)	14.67		SD (1.98)
Years of use (mean)	10.29		SD (3.10)
Total lifetime joints (mean)	4687.57		SD (3082.22)
Alcohol use in the past year (mean ± SD; *n* of days)	121.33 ± 89	85.74 ± 96.57	*t* = 1.243, *p* = 0.221
Current nicotine users (%)	57.1%	19%	Fisher’s exact = 0.043
Cocaine use (past year)			
No cocaine use in the past year (%)	57.1%	90.1%	Fisher’s exact = 0.032
Use of cocaine a few times in the past year (%)	33.3%	0%	Fisher’s exact = 0.009
Use of cocaine once or twice a month (%)	9.5%	9.5%	Fisher’s exact = 1.00
MDMA use (past year)			
No MDMA use in the last year	71.4%	90.1%	Fisher’s exact = 0.238
Use of MDMA a few times in the last year	23.8%	0%	Fisher’s exact = 0.048
Use of MDMA once or twice a month	4.8%	9.5%	Fisher’s exact = 1.00
Hallucinogen use (past year)			
No hallucinogen use in the past year	81%	100%	Fisher’s exact = 0.107
Use of hallucinogens a few times in the last year	14.3%	0%	Fisher’s exact = 0.232
Use of hallucinogens once or twice a month	4.8%	0%	Fisher’s exact = 1.00

### Task performance

**Fig. 1:**
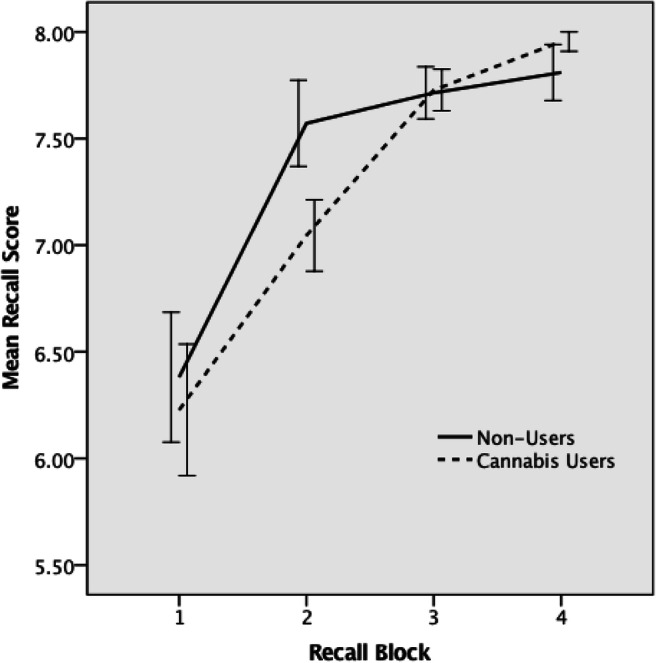
Recall mean performance over four time-points for cannabis users and non-users. Total successful recall value *p* = 0.478

There was no significant difference (*p* = 0.478) in total recall score between CU and NU. However, there was a significant effect of repetition (*p* < 0.001) on recall score, suggesting that there was a significant improvement in recall score over repeated trials across the two groups of participants. Furthermore, there was a significant interaction between group and repetition-related change in recall score such that the cubic trend in recall score over repeated trials significantly differed (*p* = 0.032, partial eta-squared = 0.108) between the CU and NU groups (Fig. 1). Post hoc comparison of the slopes using *t* test revealed that the significant interaction was mainly driven by group difference (CU vs NU) in change in recall score between blocks 2 and 3 (*p* = 0.019), but not between blocks 1 and 2 (*p* = 0.27) or between blocks 3 and 4 (*p* = 0.42). Between the groups (CU vs NU), mean recall scores were significantly different only during block 2 (*p* = 0.05), but not during blocks 1 (*p* = 0.72), 3 (*p* = 0.93) or 4 (*p* = 0.29). Furthermore, closer inspection of mean recall scores over successive blocks within each group revealed that they were significantly different in NU only between blocks 1 and 2 (*p* < 0.001), but not between blocks 2 and 3 (*p* = 0.41) or between blocks 3 and 4 (*p* = 0.49). In contrast, recall scores seemed to progressively improve in CU, such that they were significantly different between blocks 1 and 2 (*p* = 0.002), between blocks 2 and 3 (*p* < 0.001) and between blocks 3 and 4 (*p* = 0.021).

### Change in brain activation during learning over repeated encoding blocks in CU and NU

In NU, while learning word-pairs over successive encoding blocks, there were three clusters of progressive increase in brain activation with peaks in the precuneus extending to cuneus bilaterally and the right superior frontal gyrus extending to medial frontal gyrus bilaterally; and two clusters of progressive decrease in activation with peaks in the left middle frontal gyrus extending to ipsilateral superior frontal gyrus and insula; and left superior temporal gyrus extending to paracentral lobule, precentral gyrus, middle portion of the cingulate gyrus, body of caudate and inferior parietal lobule. In CU, while learning over the same successive encoding blocks, there were two clusters of progressive increase in activation with peaks in the left lingual gyrus extending to cerebellum; and right middle temporal gyrus extending to ipsilateral superior temporal and angular gyri; and two clusters of decrease in activation with peaks in the left middle frontal gyrus extending medial frontal gyrus; and right precuneus (Online Resource 3 & 4).

### Change in brain activation over repeated recall blocks in CU and NU

In NU, while recalling words over successive trials, there was a progressive increase in brain activation in the right precuneus extending to the contralateral inferior parietal lobule and a progressive decrease in activation in the left posterior part of the cingulate gyrus (Online Resource 5). No significant increase or decrease in brain activation was observed in CU over the same successive recall blocks.

### Group comparison (CU vs NU) of change in brain activation during learning over repeated encoding blocks

**Fig. 2:**
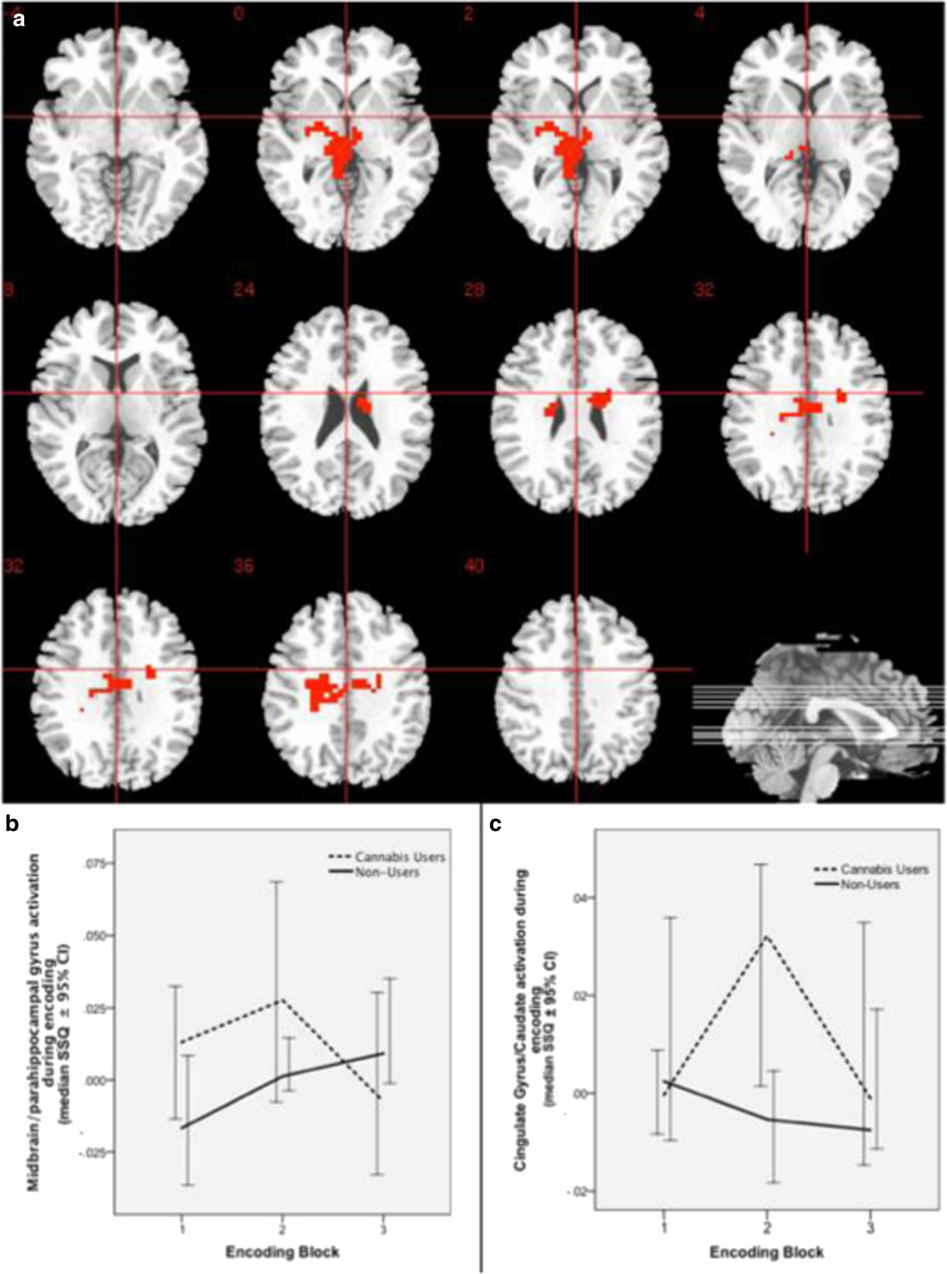
Change in brain activation during repeated encoding. **a** Group difference activation map. **b** Median activation of cannabis users and non-users in midbrain cluster extending to the parahippocampal gyrus and thalamus. **c** Median activation of cannabis users and non-users in the right cingulate gyrus extending to caudate. Right side of the brain is represented in the right side of the images

There was a significant interaction between group (CU vs NU) and learning over repeated encoding blocks in a network of brain areas that included the midbrain bilaterally extending to the parahippocampal gyrus on the left and the thalamus bilaterally (*p* = 0.00939, partial eta-squared =0.180); left cingulate gyrus and caudate extending to the insula (*p* = 0.00939, partial eta-squared = 0.100) (Table 2, Fig. 2). In the midbrain cluster extending to the parahippocampal gyrus and thalamus, there was a pattern of progressive increase in brain activation with repeated presentation of encoding blocks in NU, which was disrupted in CU. In the cingulate gyrus as well as in the body of the caudate, there was a pattern of progressive decline in activation over repeated encoding blocks in NU, while in CU there was an increase in activation from block 1 to block 2 followed by a decline over successive trials. Post hoc power analysis using effect-size estimate (partial eta-squared = 0.180; effect-size f = 0.468) from the midbrain/parahippocampal gyrus cluster found that our sample (total *N* = 42) had 84.2% power (alpha = 0.05, 2-tailed; G-POWER (Faul et al. 2007)) to detect differences in hippocampal activation between the CU and NU groups.

**Table 2:** Clusters showing change in brain activation over repeated encoding and recall trials in cannabis users and non-users

Area	*x*	*y*	*z*	Side	Cluster size	Cluster *p* value
Non-users: increase in activation over repeated encoding blocks						
Precuneus extending to cuneus	29	− 63	36	R	136	0.0032
Precuneus extending to cuneus	− 33	− 70	33	L	98	0.004
Superior frontal extending to medial frontal gyrus bilaterally	14	41	40	R	211	0.0011
Non-users: decrease in activation over repeated encoding blocks						
Middle frontal extending to ipsilateral superior frontal gyrus and insula	− 22	52	26	L	116	0.0027
Superior temporal gyrus extending to paracentral lobule, precentral gyrus, mid-cingulate gyrus, body of caudate and inferior parietal lobule	− 54	0	− 3	L	753	0.0001
Cannabis users: increase in activation over repeated encoding blocks						
Lingual gyrus extending to cerebellum	− 22	− 70	− 7	L	63	0.000665
Middle temporal gyrus extending to superior temporal and angular gyri	36	− 60	13	R	85	0.001219
Cannabis users: decrease in activation over repeated encoding blocks						
Middle frontal gyrus extending to medial frontal gyrus	− 33	37	23	L	77	0.001453
Precuneus	4	− 41	46	R	49	0.002794
Cannabis users vs non-users: change in activation over repeated encoding blocks						
Midbrain, extending to ipsilateral parahippocampal gyrus and thalamus bilaterally	− 7	− 22	− 3	L	68	0.00939
Cingulate gyrus, extending to the caudate bilaterally	22	− 4	23	R	104	0.00939
Non-users: increase in activation over repeated recall blocks						
Precuneus, extending to the left inferior parietal lobule	14	− 48	53	R	69	0.00318
Non-users: decrease in activation over repeated recall blocks						
Posterior cingulate cortex	− 18	− 56	23	L	55	0.00184
Cannabis users vs non-users: change in activation over repeated recall blocks						
Cerebellum	0	− 41	− 20	R	80	0.000068
Superior temporal gyrus, extending to ipsilateral middle temporal gyrus, caudate and posterior cingulate	− 54	− 52	10	L	60	0.001963
Insula, extending ipsilateral superior temporal gyrus	40	− 11	23	R	59	0.00176
Clusters showing change in brain activation correlating with new learning						
Activation correlating with new learning over repeated encoding blocks						
Cannabis users > non-users						
Midbrain extending bilaterally, and to the left cerebellum	− 7	− 22	− 3	L	62	0.002019
Non-users > cannabis users						
Cingulate gyrus, extending to the ipsilateral postcentral gyrus, inferior parietal lobule and tail of caudate	−4	− 15	26	L	101	0.003863
Activation correlating with new learning over repeated recall blocks						
Cannabis users > non-users						
Paracentral lobule, extending to ipsilateral precuneus	7	− 37	53	R	33	0.007386

### Change in brain activation over repeated recall blocks in CU and NU and group comparison (CU vs NU) of change in brain activation over repeated recall blocks

**Fig. 3:**
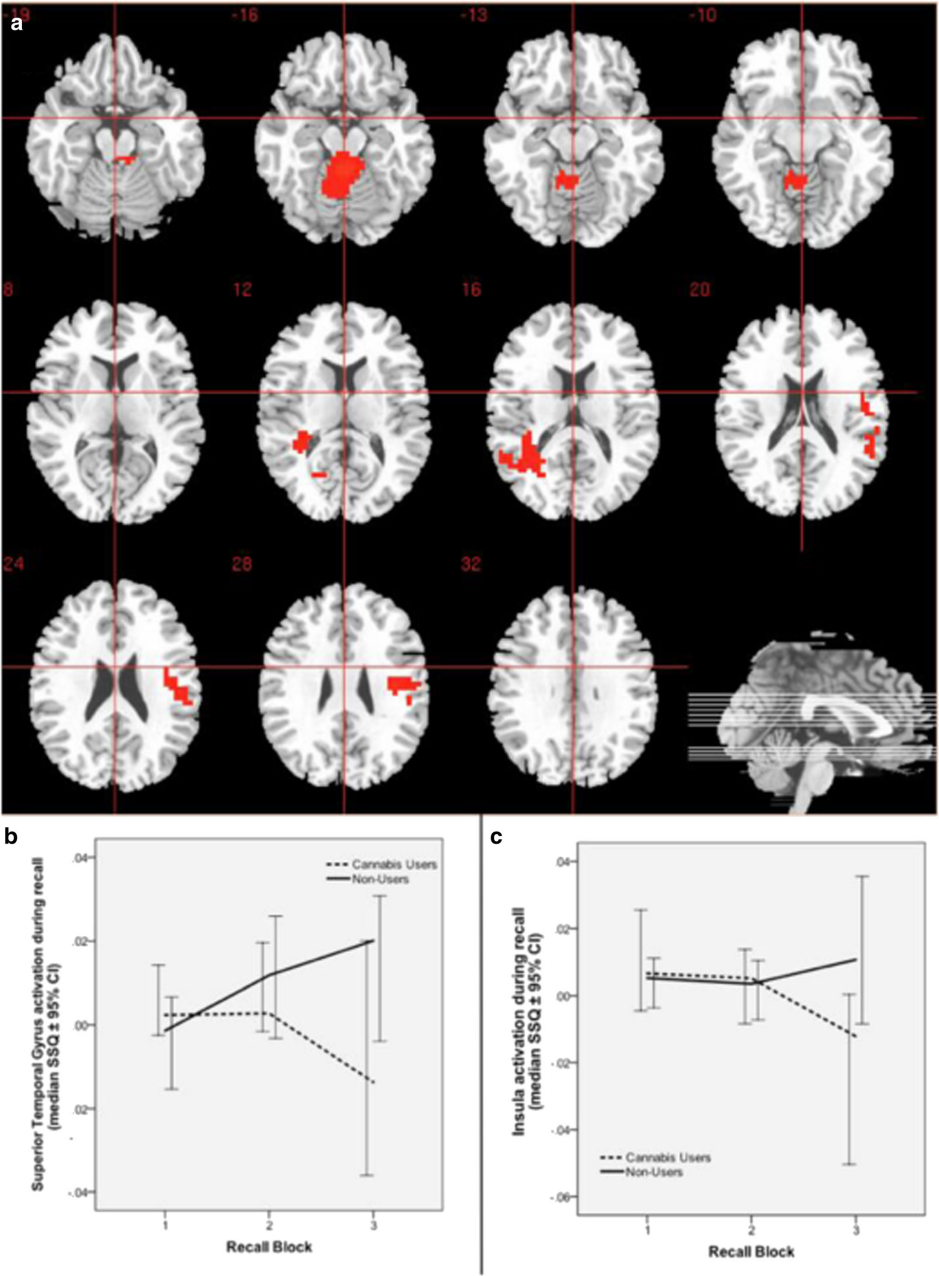
Change in brain activation during repeated recall. **a** Group difference activation map. **b** Median activation of cannabis users and non-users in the left superior temporal gyrus, extending to the middle temporal gyrus and caudate. **c** Median activation of cannabis users and non-users in the right insula extending to the superior temporal gyrus. Right side of the brain is represented in the right side of the images

There was a significant interaction between group (CU v NU) and recall over repeated recall blocks in brain regions that included the right insula extending to ipsilateral superior temporal gyrus (*p* = 0.00176, partial eta-squared = 0.005); left superior temporal gyrus extending to ipsilateral middle temporal gyrus, caudate and posterior cingulate (*p* = 0.001963, partial eta-squared = 0.064); and the cerebellum (*p* = 0.000068, partial eta-squared = 0.227) (Table 2, Fig. 3).

In the left superior temporal gyral cluster extending to the middle temporal gyrus, and caudate, there was a progressive increase in activation over successive recall blocks in NU, while in CU, activation started at the same level as NU during the first block and did not change until the 2nd recall block, declining subsequently. In the right insula cluster extending to the superior temporal gyrus, activation did not differ between the NU and CU over the first couple of recall blocks, diverging subsequently, with CU showing slight decline and NU displaying an upward trend. In contrast, there was a different pattern of change in activation in the cerebellar cluster, where NU displayed a progressive decline and CU displayed an opposite pattern over successive recall blocks.

### Relationship between repetition-related change in brain activation and incremental novel learning

#### Encoding

Direct comparison of the association between change in brain activation over repeated encoding blocks and incremental novel learning (as indexed by the ‘new learning score’) over the same encoding blocks confirmed the learning-related differential pattern of activation in the two groups. CU showed significantly greater correlation than NU between repetition-related change in activation during encoding and the number of successfully learned new word-pairs over the same successive encoding blocks in the midbrain bilaterally, extending to the left parahippocampal gyrus, culmen and thalamus. NU showed significantly greater correlation than CU in the left cingulate gyrus, extending to the ipsilateral postcentral gyrus, inferior parietal lobule and tail of caudate (Table 2, Online Resource 6).

#### Recall

Direct comparison of the association between change in brain activation over repeated recall blocks and incremental novel learning (as indexed by the ‘new learning score’) over the same recall blocks showed significantly greater correlation in CU than in NU between repetition-related change in activation during recall and the number of successfully recalled new word-pairs over the same successive recall blocks in the right paracentral lobule extending to ipsilateral precuneus (Table 2, Online Resource 6). There were no regions where correlation of brain activation and incremental novel learning was greater in NU than in CU.

#### Confounding effect of other drug use

No difference was seen in the results for repeated encoding or recall after covarying for cocaine, MDMA, hallucinogenic and nicotine use separately and collectively. Participant’s other drug use data are shown in Table 1.

## Discussion

The main focus of this study was to investigate the neurophysiological abnormalities that may underlie impairments in verbal learning and memory in regular cannabis users. Using a paired associate verbal learning task in conjunction with fMRI and an analytic approach that allowed us to investigate progressive changes in learning-related engagement of different brain regions, we show that CU had a slower learning trajectory and employed a significantly different pattern of recruitment of brain regions relative to NU while learning word-pairs. The verbal learning task employed had a relatively modest level of difficulty, such that performances in both the CU and NU groups reached ceiling and were not significantly different as indexed by their total recall score. Nevertheless, the gradient of learning across the repeated trials was significantly different between NU and CU. NU seemed to learn the word-pairs faster than CU, such that performance in NU improved significantly from recall block 1 to reach near the ceiling by recall block 2, with no significant further improvement as indicated by lack of significant difference in recall scores between blocks 2 and 3 and between blocks 3 and 4. In contrast, CU seemed to continue to learn with progressive improvement in recall score over successive trials until block 4, reaching a similar performance level as NU only by recall block 3. While learning the verbal stimuli over repeated encoding blocks, NU showed a progressive increase in recruitment of a cluster of brain regions that included the midbrain, parahippocampal gyrus and thalamus, which paralleled the progressive improvement in total number of words learnt per block as indexed by subsequent recall. However, this pattern of progressive increase in recruitment of these brain regions was disrupted in CU, who engaged these regions to a greater extent than NU to start with. Furthermore, this was associated with the recruitment of additional regions. Progressive change in midbrain/parahippocampal function over successive encoding blocks showed a stronger correlation with new word-pairs learnt over the same blocks in CU than in NU suggesting that slower verbal learning in CU was a consequence of disrupted parahippocampal and midbrain function. CU seemed to compensate for this by engaging the cingulate cortex and caudate to a greater extent than in NU during encoding to attain the same level of subsequent recall performance.

To the best of our knowledge, none of the published studies to date have compared trial-by-trial learning-related change in brain activation in CU with NU. Three studies have investigated brain activation while learning during encoding condition of associative memory tasks in adult CU (Carey et al. 2015; Jager et al. 2007; Nestor et al. 2008). As indicated earlier, all of them reported altered medial temporal activation in region of interest analyses, though there was no consistent direction of change in relation to NU (Carey et al. 2015; Jager et al. 2007; Nestor et al. 2008). This may reflect reported differences between the studies in terms of methodology (such as duration of abstinence or specific contrast examined during analysis) as well as task performance difference between CU and NU. However, it is worth noting that greater parahippocampal engagement in CU than in NU during the initial blocks of learning in the present study is comparable to that observed during learning across all encoding blocks independent of repetition by Nestor and colleagues (Nestor et al. 2008), the study that perhaps adopted a design closest to that in the present study in terms of consideration of duration of abstinence. Our results are also consistent with a previous report of greater activation in CU than in NU in the cingulate gyrus and caudate before the encoding condition of a spatial working memory task (Kanayama et al. 2004), in the absence of group difference in task performance. Altered activation in these regions has also been reported in studies that employed cognitive activation paradigms that did not involve memory processing (Block et al. 2000; Gruber et al. 2009; Harding et al. 2012; Hester et al. 2009; van Hell et al. 2010).

While we had predicted that altered medial temporal activation during repeated learning trials would differentiate CU from NU, we observed that these groups also differed in learning-related engagement in a number of other brain regions. Although the medial temporal cortex is critical for learning new information (Schacter and Wagner 1999), growing evidence also supports a key role for the midbrain in supporting the encoding and updating of contextual information in memory (D'Ardenne et al. 2012; Murty et al. 2011; Schott et al. 2006; Schott et al. 2004). Co-activation of the medial temporal cortex and midbrain during verbal learning has also been shown to be important for the integration of separate learning events into a linked mnemonic representation (Shohamy and Wagner 2008; Zeithamova et al. 2016) and promote new learning (Zeithamova et al. 2016). Results of the present study extend previous evidence to show that regular cannabis use may disrupt the normal updating and integration of the word-pair associations over repeated trials into a mnemonic representation, and suggest that this may underlie impaired performance in memory tasks (Grant et al. 2003; Meier et al. 2018; Schreiner and Dunn 2012) in CU. Greater activation in the dorsal anterior cingulate and the striatum in CU than in NU may reflect greater monitoring (Cabeza and St Jacques 2007; Fleck et al. 2006; Simons et al. 2005) of task performance and compensatory striatal engagement to support the encoding and updating of contextual information (Dahlin et al. 2008; Landau et al. 2009; Lewis et al. 2004; Murty et al. 2011) respectively in CU, which may have helped ensure that their total recall score across all blocks was comparable to NU.

CU and NU also differed in brain activation over repeated recall blocks. Progressive improvement in recall performance in NU was associated with a progressive increase in engagement of the left superior and middle temporal gyri and ipsilateral caudate and posterior cingulate over the corresponding recall blocks. In contrast, this incremental pattern of recall-related brain activation was disrupted in CU. CU seemed to compensate for this by engaging the cerebellum to a progressively greater extent over the corresponding recall blocks. Reduced or decremental pattern of activation in the lateral temporal and posterior cingulate cortex, insula and caudate in CU in contrast to the incremental pattern of activation observed in these regions, which are typically engaged during semantic retrieval (Spaniol et al. 2009), in NU may underlie slower improvement in trial-by-trial recall score in CU.

The insula, caudate and cingulate gyrus are also key components of the salience network (Menon and Uddin 2010) and support the switch from the default mode network to the central executive network while performing a cognitive task (Menon 2011; Sridharan et al. 2008). Therefore, incremental pattern of engagement of this region during cued recall may underlie progressive improvement in recall performance in NU. In contrast, decremental pattern of activation in these regions was observed in CU, which may underlie their slower improvement in trial-by-trial recall score. Greater cerebellar activity during repeated recall trials in CU than in NU may have helped compensate for this (Davachi et al. 2001; Guell et al. 2018; Paulesu et al. 1993; Stoodley and Schmahmann 2009) and may reflect progressively greater demand for working memory resources (Desmond et al. 1998) in CU to support cued recall over successive trials.

Results reported here need to be considered in light of certain limitations. While our analytic approach allowed us to investigate trial-by-trial changes in brain activation during learning, it may be argued that these changes represent non-specific changes in attention, or adaptive changes as a result of repeated presentation of the same stimuli (Larsson et al. 2016). However, further analyses showed that repetition-related differential pattern of change in activation in the midbrain, parahippocampal cortex and thalamus in the two groups were associated with progressive change in new word-pairs learnt over successive trials. It may also be argued that differences in brain activation observed between CU and NU may not reflect the effects of cannabis use alone, but instead may be confounded by group differences in comorbid exposure to other drugs. However, when we controlled for these group differences in cocaine, MDMA, hallucinogenic and nicotine use, the direction and pattern of our key findings did not change. Nevertheless, we cannot completely rule out such effects. While a study investigating functional differences between CU and NU without any comorbid use of other psychoactive substances would be ideal, such participants are perhaps less representative of the general population of regular cannabis users. It is also worth noting that CU were asked to refrain from cannabis use from the night before scanning to avoid acute effects of the drug, as in other fMRI studies (Gruber et al. 2009; Sneider et al. 2013). Acute psychotropic effects of cannabis are believed to peak 1–2 h after administration (Bhattacharyya et al. 2010), while THC may be present in the body up to 1 month after ingestion, with the half-life for regular users being around 5–13 days (Sharma et al. 2012). Therefore, some of the group differences observed may reflect the effects of any residual THC present in CU. Whether the functional alterations observed here persist after a sustained period of abstinence such that any residual THC has completely washed out of the body warrant further investigation. It is also important to note that we cannot be completely certain that the differences observed between CU and NU reflect the effects of cannabis use on brain functioning as opposed to underlying differences between these two groups of participants that may have predisposed CU to cannabis use. This is a limitation of cross-sectional studies such as this, and longitudinal or twin/sibling designs (Paul and Bhattacharyya 2018) are necessary to disentangle whether differences observed are a cause or consequence of use.

An important methodological aspect of the present study was the requirement that all cannabis-using participants should have started smoking cannabis regularly before the age of 18 years. The period of adolescence represents a critical period of vulnerability to exogenous insults, and is when many developmental processes including brain development and binding affinity of cannabinoid 1 receptors, the target of ingredients in recreational cannabis in the brain, are in a state of flux before attaining maturity in early adulthood (Andersen 2003; Belue et al. 1995; Rice and Barone 2000; Spear 2007). Preclinical research has also shown adolescence to be a period of greater sensitivity to THC (Quinn et al. 2008; Schneider and Koch 2003), and that starting to use cannabis during this period has greater detrimental effects (Pope et al. 2003; Wilson et al. 2000). Therefore, this approach allowed us to examine the effects of cannabis use that started at a time when the brain is most vulnerable to its detrimental effects. In this context, it may be argued that the threshold that we have employed for defining adolescent-onset use (i.e. use more than twice per month and at least 10 times use in lifetime, before the age of 18) may not truly reflect ‘regular’ use before adolescence. However, it is worth noting that this threshold was based on a previous study that surveyed cannabis use patterns amongst adolescents from 31 countries (Sznitman et al. 2015), and therefore is likely to be representative of usage pattern around initiation of cannabis use in typical adolescent-onset users. While the minimum use threshold before the age of 18 years was set as above, in reality, 20 out of the 21 cannabis-using participants in our study were using cannabis at least once a week before the age of 18, with the remaining participant having used several times a month by that age. Furthermore, it is worth noting that for inclusion into the study, these thresholds were used only for the determination of usage pattern before the age of 18 and additional inclusion criteria required use at least 4 times per week for at least 2 years before taking part in the study. Another issue worth noting is that we investigated both males and females and did not examine the effects of sex. While the role of sex as a potential factor moderating the effects of long-term cannabis use has been proposed in previous literature, we did not investigate this as the present study was not designed, and hence not powered to examine this. However, this clearly warrants examination in future studies.

What might this mean in terms of our understanding of the neurocognitive mechanisms that may underlie the association between cannabis use and schizophrenia? Impairments in verbal learning and memory have long been associated with schizophrenia (Aleman et al. 1999), as well as structural (Nenadic et al. 2015) and functional changes (Mwansisya et al. 2017) in a number of neural substrates linked to verbal memory (Guimond et al. 2016). Abnormal medial temporal function especially during memory tasks is one of the key alterations reported in schizophrenia (Heckers 2001) and onset of psychosis has been linked to alterations in medial temporal structure (Mechelli et al. 2011) and function (Allen et al. 2017; Allen et al. 2016; Allen et al. 2012) and abnormal dopamine function in the midbrain. As a growing body of experimental, epidemiological and clinical evidence point towards associations between acute exposure to psychoactive cannabinoids and transient psychotic symptoms (Bhattacharyya et al. 2009) and continued cannabis use and increased risk of onset and relapse of schizophrenia (Gage et al. 2016; Kraan et al. 2016; Sami and Bhattacharyya 2018; Schoeler et al. 2016a; Schoeler et al. 2016b; Schoeler et al. 2016c), results presented here could suggest potential neurocognitive mechanisms through which cannabis use may increase the risk of development of schizophrenia.

The precise neurochemical mechanisms underlying the functional alterations observed in CU are unclear. Alterations in both dopaminergic (Bloomfield et al. 2014; van de Giessen et al. 2017) (also reviewed in (Sami et al. 2015)) and glutamatergic (Colizzi et al. 2016) function in relation to cannabis use have been reported, though their relationship with functional alterations has yet to be investigated.

In summary, here, we report evidence of disrupted medial temporal and midbrain function underlying slower learning in adolescent-onset cannabis users. Future studies should investigate whether these functional alterations may underlie the increased risk of psychosis associated with regular cannabis use.

## Electronic supplementary material

ESM 1 (PDF 1640 kb)
