# Supplementary material for: Disrupted parahippocampal and midbrain function underlie slower verbal learning in adolescent-onset regular cannabis use
Source: Psychopharmacology (Berl). 2019 Dec 9;238(5):1315–31. doi: 10.1007/s00213-019-05407-9 (PMC8062355; doi:10.1007/s00213-019-05407-9)
Supplement: Supplementary file 1 — (PDF 1640 kb) [file 213_2019_5407_MOESM1_ESM.pdf]

Online Resource

Disrupted parahippocampal and midbrain function underlie slower verbal learning in adolescent-onset regular cannabis use

Authorline: Grace Blest-Hopley[1], Aisling O'Neill[1], Robin Wilson[1,] Vincent Giampietro[2], Sagnik Bhattacharyya[1, 3]*

[1] Department of Psychosis Studies, Institute of Psychiatry, Psychology & Neuroscience, King's College London, UK.

[2] Department of Neuroimaging, Institute of Psychiatry, Psychology & Neuroscience, King's College London, UK.

[3] South London and Maudsley NHS Foundation Trust, Denmark Hill, Camberwell, London, UK

*Corresponding author

Department of Psychosis Studies, Institute of Psychiatry, Psychology & Neuroscience, King's College London, De Crespigny Park, London SE5 8AF, UK, Tel +44 207 848 0955, Fax +44 207 848 0976, Email: sagnik.2.bhattacharyya@kcl.ac.uk

Online Resource 1

Supplementary Methods

Brain activation across all encoding and recall blocks independent of repetition: group comparison between cannabis users and non-users

Comparison of regional brain activation between cannabis users and non-users was carried out using whole brain analysis of variance (ANOVA) for each of the two task conditions (encoding and recall) independent of repetition. The voxel-wise statistical threshold was set at $p=0.05$ and the cluster-wise thresholds were adjusted to ensure that the number of false positive clusters per brain would be <1 (only regions that survived this critical statistical threshold are reported).

Supplementary Results

Group differences in brain activation during the encoding and recall conditions of the verbal memory task independent of repetition

At the whole brain level, CU were found to have increased activation compared to NU in the superior, inferior and middle frontal gyri bilaterally and the right medial frontal gyrus during the encoding condition of the verbal memory task. CU did not show any regions of decreased activation compared to NU.

There were no significant differences in brain activation between CU and NU during the recall condition of the memory task.

Online Resource 2

Supplementary Table 1: Activation during verbal learning task independent of repetition						
Encoding condition						
Cannabis Users > Non-Users						
Voxel Size	x	y	z	p	Side	
92	43	19	10	0.000677	Right	Inferior frontal gyrus extending to the superior, middle and medial frontal gyrus.
95	-22	52	17	0.00058	Left	Superior frontal gyrus extending to the middle and inferior frontal gyrus.
Recall condition						
No clusters showing significant differences in activation between cannabis users and non-users.						

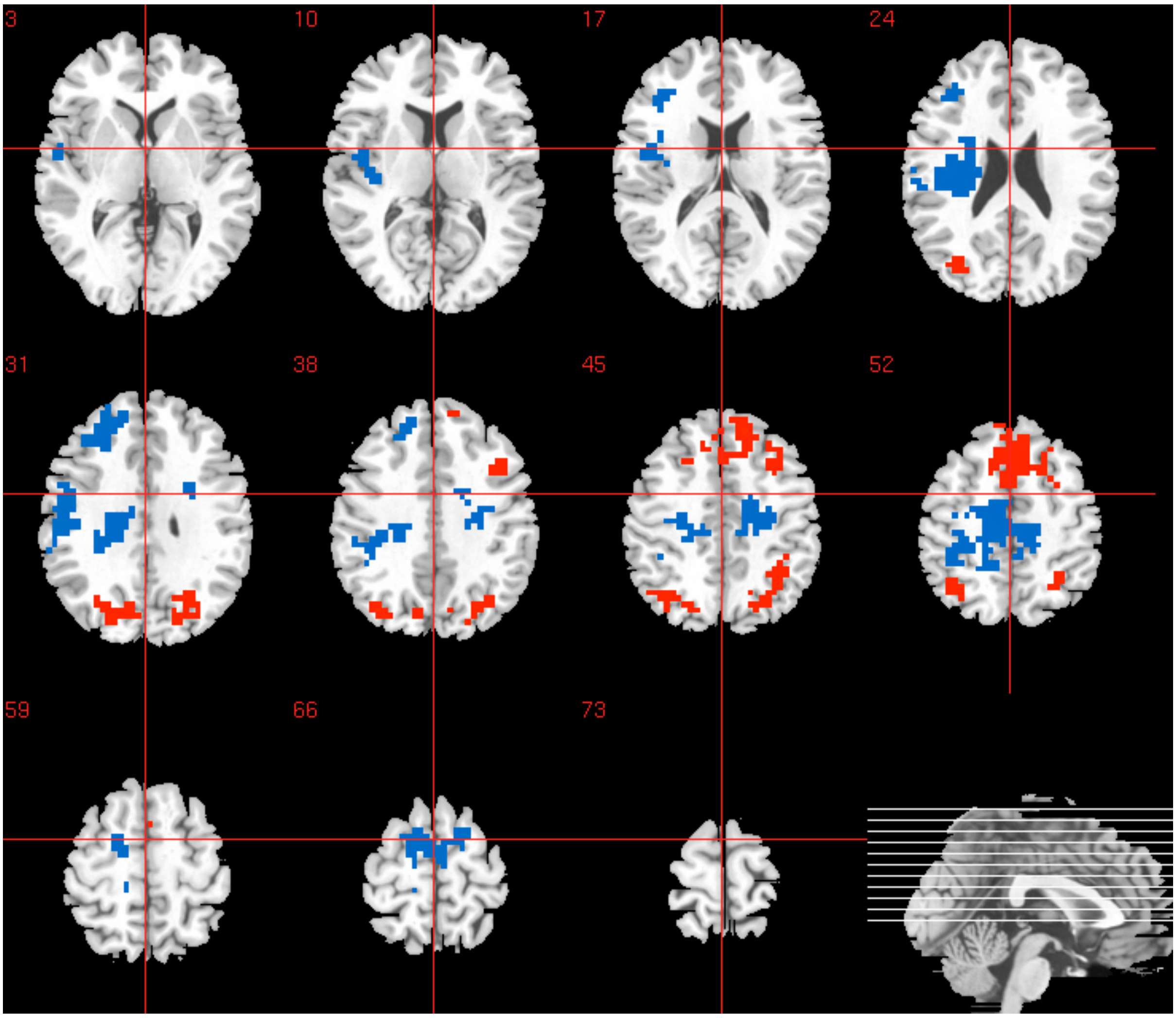

Online Resource 3 Change in brain activation in NU over repeated encoding trials. Progressive increase in activation over repeated encoding blocks shown in red, and progressive decrease in activation over the same blocks shown in blue. Right side of the brain is represented in the right side of the images

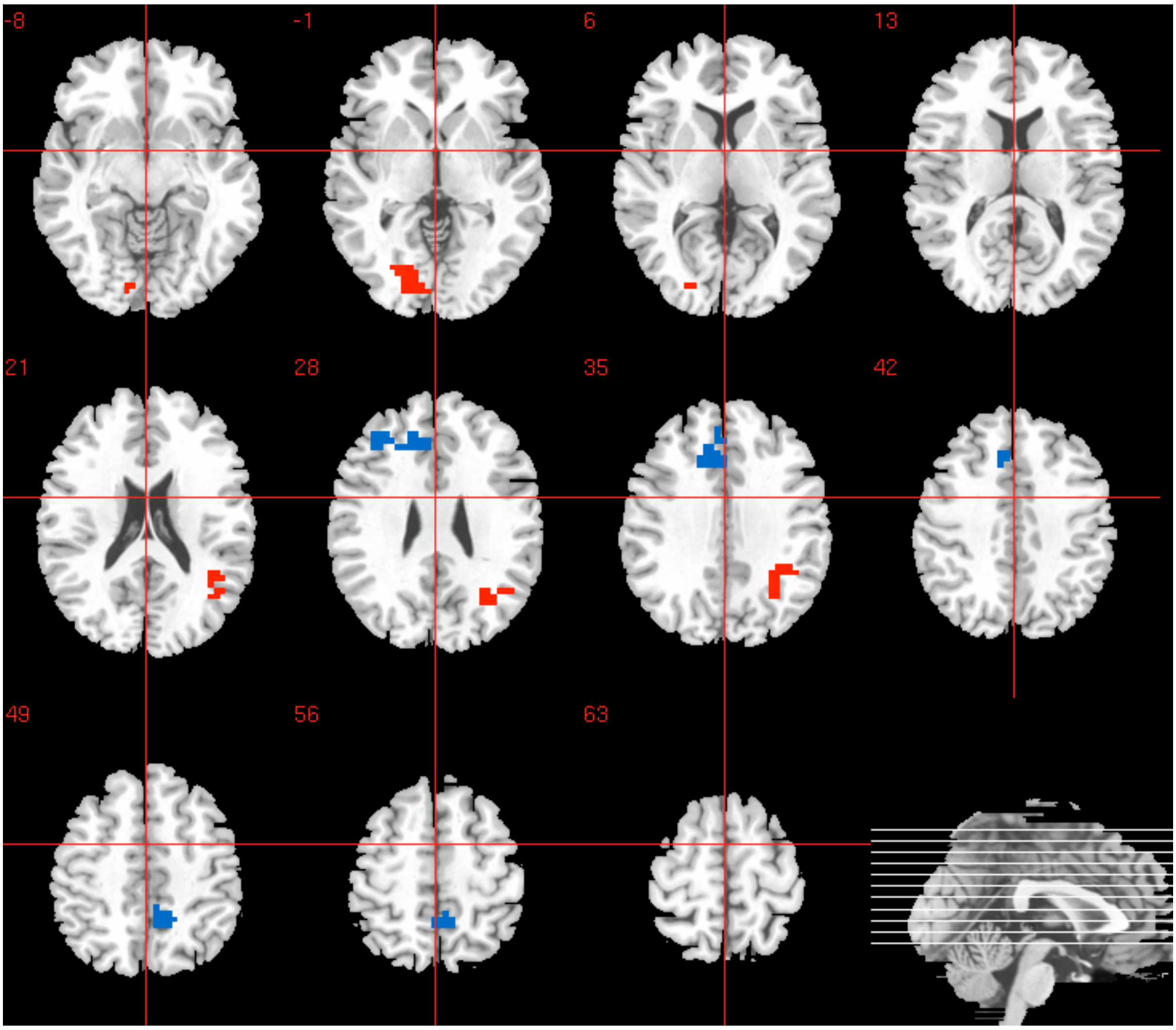

Online Resource 4 Change in brain activation in CU over repeated encoding trials. Progressive increase in activation over repeated encoding blocks shown in red, and progressive decrease in activation over the same blocks shown in blue. Right side of the brain is represented in the right side of the images

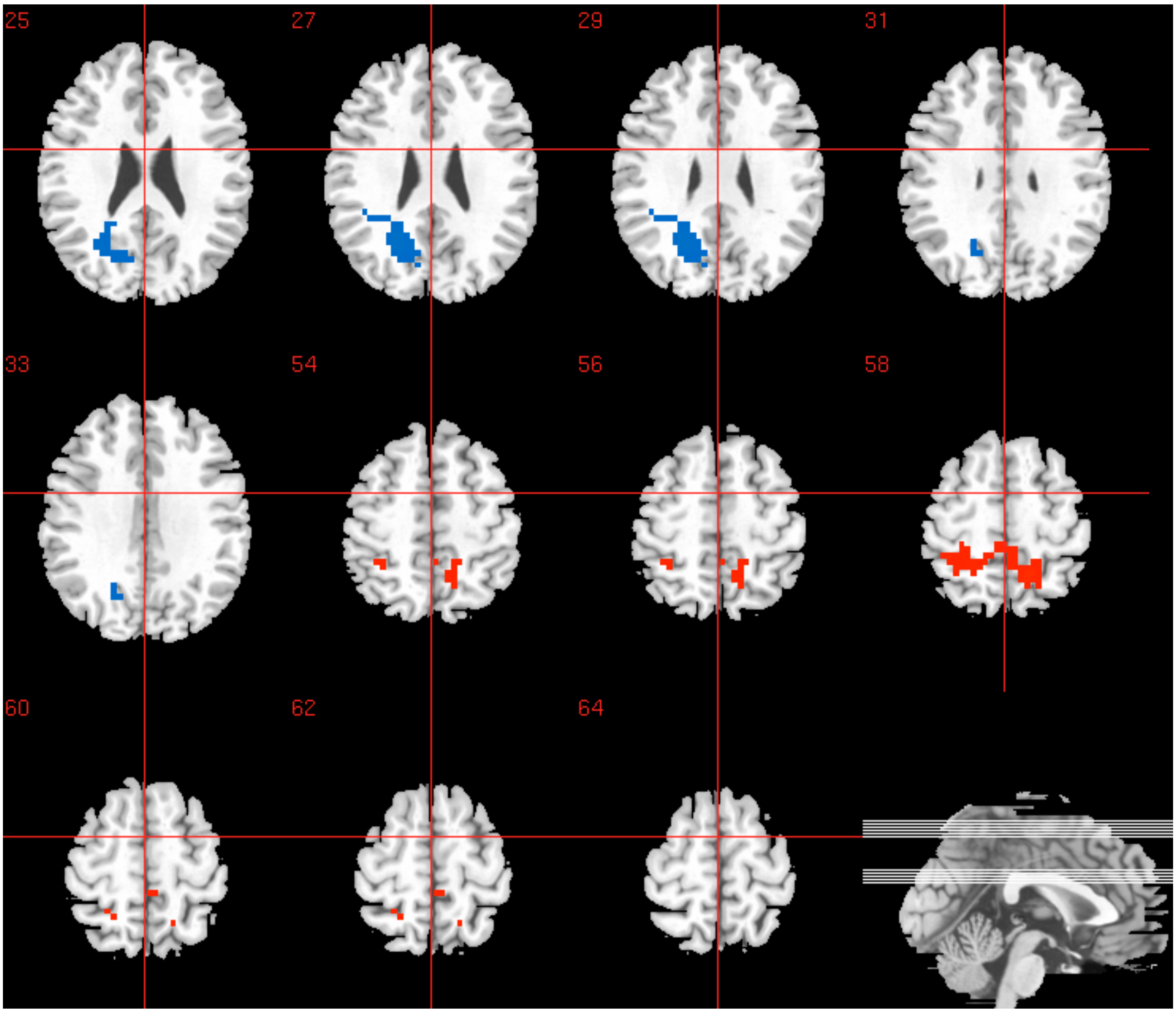

Online Resource 5 Change in brain activation in NU over repeated recall trials. Progressive increase in activation over repeated recall blocks shown in red, and progressive decrease in activation over the same blocks shown in blue. Right side of the brain is represented in the right side of the images

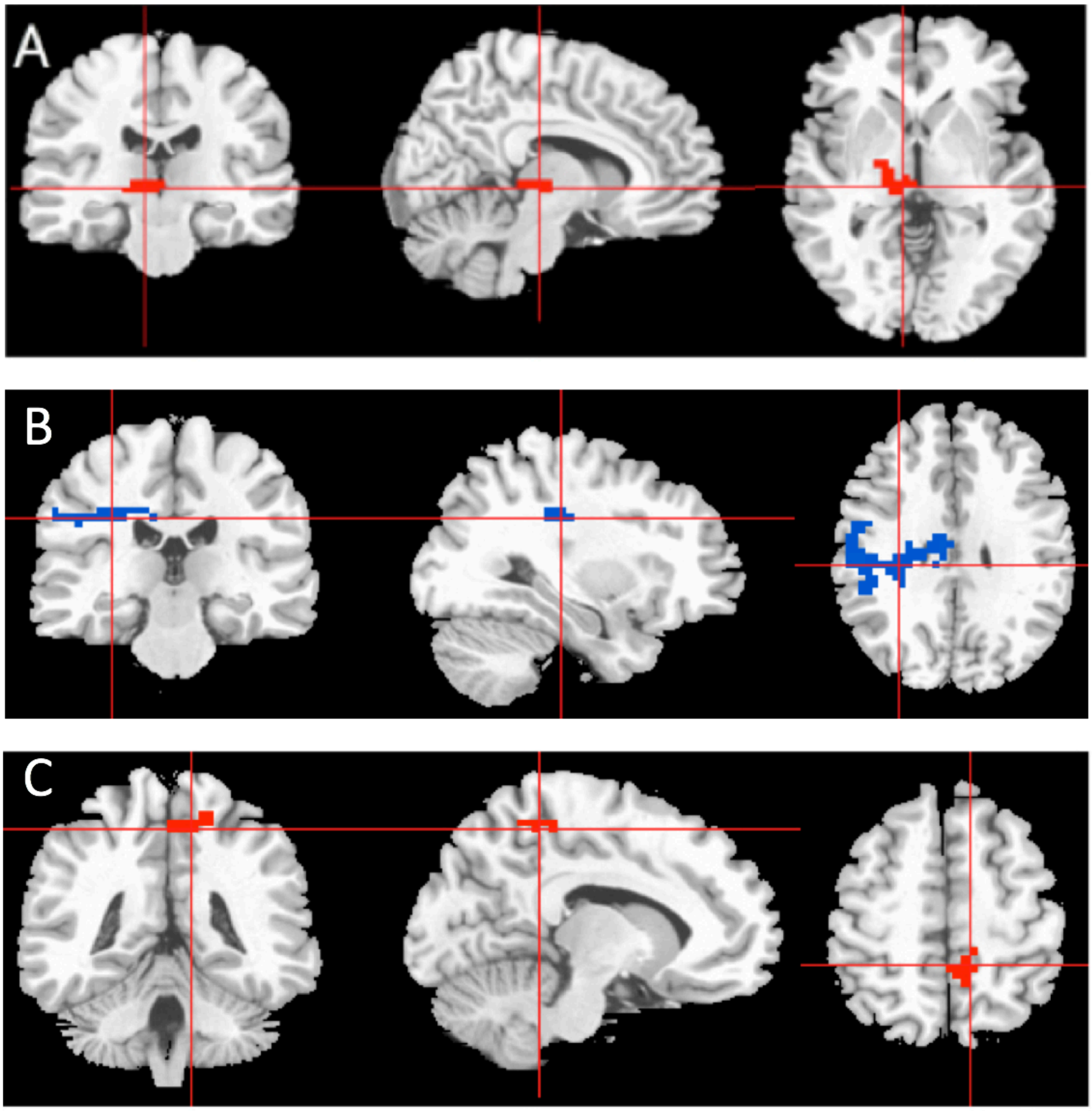

Online Resource 6 Clusters showing change in brain activation correlating with trial-by-trial new learning **A** - Change in brain activation over repeated encoding blocks showing greater correlation in CU than in NU with trial-by-trial new learning **B** - Change in brain activation over repeated encoding blocks showing greater correlation in NU than in CU with trial-by-trial new learning **C** - Change in brain activation over repeated recall blocks showing greater correlation in CU than in NU with trial-by-trial new learning; Right side of the brain is represented in the right side of the images
